# Ginsenoside Rg1 attenuates isoflurane/surgery-induced cognitive disorders and sirtuin 3 dysfunction

**DOI:** 10.1042/BSR20190069

**Published:** 2019-10-15

**Authors:** Hui-Hui Miao, Min Wang, Hai-Xia Wang, Ming Tian, Fu-Shan Xue

**Affiliations:** Department of Anesthesiology, Beijing Friendship Hospital, Capital Medical University, Beijing, People’s Republic of China

**Keywords:** Ginsenoside Rg1, Mitochondrion dysfunction, Oxidation stress, Perioperative neurocognitive disorders, Sirtuin3

## Abstract

Isoflurane/surgery (I/S) may induce neurocognitive disorders, but detailed mechanisms and appropriate treatment remain largely unknown. This experiment was designed to determine whether ginsenoside Rg1 could attenuate I/S-induced neurocognitive disorders and Sirtuin3 (Sirt3) dysfunction. C57BL/6J male mice received 1.4% isoflurane plus abdominal surgery for 2 h. Ginsenoside Rg1 10 mg/kg was intraperitoneally given for 8 days before surgery. Neurocognitive function was assessed by the Barnes Maze test. Levels of reactive oxygen species (ROS), oxygen consumption rate (OCR), mitochondrial membrane potential (MMP), expression and deacetylation activity of Sirt3 in the hippocampus tissues were measured. Results showed that I/S induced hippocampus-dependent learning and memory impairments, with increased ROS levels, and reduced OCR, MMP, and expression and deacetylation activity of Sirt3 in hippocampus tissues. Ginsenoside Rg1 treatment before I/S intervention significantly ameliorated learning and memory performance, reduced ROS levels and improved the OCR, MMP, expression and deacetylation activity of Sirt3. In conclusion, this experiment demonstrates that ginsenoside Rg1 treatment can attenuate I/S-induced neurocognitive disorders and Sirt3 dysfunction.

## Introduction

Perioperative neurocognitive disorders (PNDs) are the common complications of surgical patients, with deterioration in memory, attention and speed of information processing [1]. It has been shown that PNDs are significantly associated with poor short- and long-term postoperative outcomes [2]. As the precise pathogenesis of PNDs is not known, there is still the lack of effective treatments.

Mitochondria are the essential organelles for energy production, and regulation of signaling cascades and cell death. Recently, it has become apparent that neurodegenerative diseases are associated with mitochondrial dysfunction [3]. The studies have indicated the role of mitochondrial pathway in the neurocognitive disorders induced by surgery and volatile anesthetics [4–7]. Especially, anesthetics may influence mitochondrial size and structural integrity. It is generally believed that increased reactive oxygen species (ROS) may cause unbalance of mitochondrial fission and fusion. Furthermore, declined mitochondrial membrane potential (MMP) may cause the opening of mitochondrial permeability transition pore (mPTP), then aggravating the generation of ROS [7]. Most important, the MMP decline may be due to the dysfunction of Sirtuin3 (Sirt3), a key compound to regulate mitochondrial energy metabolism and oxidative stress. The available evidence indicates that Sirt3 is involved in cognition decline of Alzheimer’s disease [8]. In addition, deficiency of Sirt3 may increase the acetylated-cyclophilin D and ROS levels to exacerbate the mitochondrial dysfunction [9].

Ginseng, the root of Panax ginseng C. A. Meyer (Araliaceae family), has been used in traditional Chinese medicine for a long time and all over the world [10]. Ginsenosides are the main active components of ginseng with different pharmacological effects. At present, more than 60 ginsenosides have been identified and can be divided into three types: A-Panaxadiol group (e.g. Rb1, Rb2, Rb3, Rc, Rd, Rg3 and Rh2), B-Panaxatriol group (e.g. Re, Rg1, Rg2 and Rh1) and C-Oleanolic acid group (e.g. Ro) [6,11]. Among these, ginsenoside Rg1 has been used for treatment of central nervous system dysfunctions, especially those involving cognitive abilities such as learning and memory [12]. Ginsenoside Rg1 has been shown to attenuate oxidative damage by inhibiting lactate dehydrogenase efflux, nitric oxide production, ROS induction and lipid peroxidation in the Alzheimer’s disease model. Furthermore, Rg1 treatment can inhibit the Aβ-induced increases in caspase-3 activity, phosphorylated tau and activation of p38 MAPK [13,14]. Our previous work in H4-naïve and H4-APP cells has shown that ginsenoside Rg1 can protect isoflurane-induced neuron apoptosis [6]. However, it is unclear whether ginsenoside Rg1 can provide a protection against isoflurane/surgery (I/S)-induced neurocognitive disorders. Thus, this experiment was designed to assess whether ginsenoside Rg1 could attenuate I/S-induced neurocognitive disorders and explore the Sirt3-related mitochondrial mechanisms in mice.

## Materials and methods

### Animals and experiment protocol

After the protocol was approved by the Ethical Committee of Beijing Friendship Hospital, Capital Medical University (Ethical Approval Number: 18-2003), wild-type C57BL/6J mice (4–5-month-old, male) were employed. In the present study, only male mice were used to reduce the potential influences of fluctuating serum estrogen and progesterone concentrations on learning and memory in female mice. The mice were group-housed with four mice per cage. All procedures were performed in accordance with the National Institutes of Health guide for the care and use of Laboratory animals (NIH Publications No. 8023, revised 1978).

Ginsenoside Rg1 was obtained from the National Institutes for Food and Drug Control (Beijing, China) in the form of white powder-like crystals, with a molecular weight of 800, general formula C_42_H_72_O_14_ and a purity of 98% or more [15]. Ginsenoside Rg1 or normal saline (NS) was given via intraperitoneal injection daily for 8 days (7 days before surgery day and then 30 min before surgery on the procedure day). The dose of ginsenoside Rg1 (10 mg/kg, daily) was selected according to the previous study [11].

This experiment was completed in the Animal Research Center of Beijing Friendship Hospital. After the mice were pretreated with ginsenoside Rg1 (Rg1) or NS, they were randomly assigned to the I/S group or control group. Therefore, there were four groups in the present study: control+NS group, control+Rg1 group, I/S+NS group and I/S+Rg1 group. The mice in I/S+NS and I/S+Rg1 groups were performed with simple laparotomy under isoflurane anesthesia, as the methods previously described [4]. Specifically, anesthesia was induced and maintained with 1.4% isoflurane in 100% oxygen in a transparent acrylic chamber. Fifteen minutes after isoflurane anesthesia, mouse was moved out of the chamber, and then anesthesia was maintained via a cone device. One 16-gauge needle was inserted into the cone near the nose of the mouse to monitor the concentration of isoflurane. A longitudinal midline incision was made from the xiphoid to 0.5 cm proximal pubic symphysis on the skin, abdominal muscles and peritoneum. Then, the incision was sutured layer by layer with 5-0 Vicryl thread. A total of 2.5% lidocaine cream was applied to relieve the wound pain, with three times per day for 2 days after surgery.

### Behavior test

**Figure 1:**
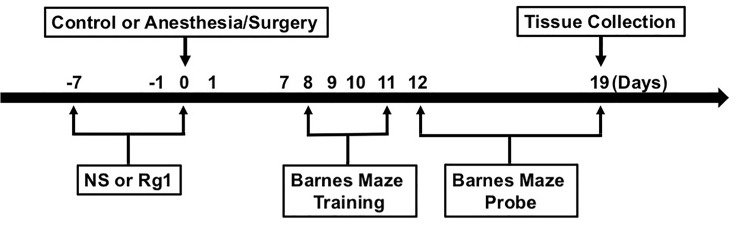
The diagram of experiment process

The Barnes maze test was performed using the methods described in other studies [16,17] with modifications. The timeline of experiment is shown in Figure 1. Six days after being exposed to various experimental treatments, animals were subjected to the Barnes maze test to assess their spatial learning and memory function. From days 8 to 11 after surgery, animals were trained in a spatial acquisition phase for 4 days, with a duration of 3 min per trial, two trials per day and 15 min between trials. The reference memory was tested on days 12 and 19 after surgery. Each animal had one trial on each of these 2 days. No test was performed during the period from days 12 to 19. Both the latency to find the escape box and the number of error holes searched during each trial were recorded as escape latency and number of errors with the assistance of ANY-Maze video tracking system. The prolongation of escape latency and the increase in number of errors suggest neurocognitive disorders of tested animals [16,17]. After each test, the Barnes maze was cleaned with 75% alcohol solution to avoid olfactory cues.

### Western blotting for Sirt3 expression

The hippocampus tissues were harvested at day 19 after surgery. The harvested hippocampus tissues were homogenized on ice using immunoprecipitation buffer (10 mmol/l Tris/HCl, pH 7.4, 150 mmol/l NaCl, 2 mmol/l EDTA, 0.5% Nonidet P-40) plus protease inhibitors (1 mg/ml aprotinin, 1 mg/ml leupeptin, 1 mg/ml pepstatin A). The lysates were collected, centrifuged at 12000 rpm for 15 min and quantified for total proteins by bicinchoninic acid protein assay kit (Pierce, Iselin, NJ), as described in our previous study [6]. Then, anti-Sirt3 (1:1000, #5490S, Cell Signaling, Danvers, MA) was used for Western blotting. The band intensity was quantified using Quantity One Analysis Software, and expressions of proteins were acquired by standardizing the gray levels of Sirt3 with β-actin as described in our previous work [5].

### ROS measurement

The ROS levels in the hippocampus tissues were measured by an OxiSelect In Vitro ROS/RNS Assay Kit (Cell Biolabs, San Diego, CA), according to the protocols provided by the manufacturer and the methods previously described in other study [4].

### Oxygen consumption measurement

Oxygen consumption rate (OCR) was measured using a Seahorse Biosciences XF Extracellular Flux Analyzer (XFe Analyzer) [18]. Mitochondria were isolated from the hippocampus tissues by mitochondria isolation kit for tissues (Thermo Scientific, U.S.A.), diluted with assay solution (KCl, KH_2_PO_4_, MgCl_2_, HEPES, EGTA and FA-free BSA), and transferred into XF24 cell culture plate. One hour before initiation of measurement, medium was replaced with XF medium supplemented with 10 mM glucose or 1 mM pyruvate and incubated for 1 h in a 37°C incubator (without CO_2_). Three baseline OCR measurements were performed, followed by injection with oligomycin (1 μM) to measure the ATP-linked OCR. The uncoupler carbonyl cyanide-4-(trifluoromethoxy) phenylhydrazone (0.5 μM) was used to determine maximal respiration, and both rotenone (1 μM) and antimycin A (1 μM) were injected to determine the non-mitochondrial respiration. Experimental treatments were performed on three wells of each plate as technical replicates and each experiment had at least three biological replicates. OCR was normalized for the amount of protein in each well [19].

### MMP and Sirt3 deacetylation activity measurements

The mitochondrial isolation from fresh hippocampus tissues was performed using the mitochondrial isolation kit (Thermo Fisher Scientific, Waltham, MA, U.S.A.). According to the manufacturer’s instructions, the hippocampus tissues were homogenized using glass homogenizer within mitochondrial isolation buffer, then centrifuged at 700×***g*** for 10 min at 4°C, and the supernatant was centrifuged at 3000×***g*** for 15 min. The pellets were used for MMP and Sirt3 deacetylation activity freshly.

The MMP was determined by the JC-1 MMP detection kit (Biotium, Hayword, CA, U.S.A.), as to the methods previously described in other study [20] and the manufacturer’s instructions. The Sirt3 deacetylation activity was measured using the Sirt3 activity assay kit (#ab156067, Abcam, Cambridge, MA), following the protocols provided by the manufacturer and method previously described in other study [8].

### Statistics analysis

Data were expressed as mean ± standard error of mean (SEM). The differences among groups were assessed by the two-way ANOVA, followed by Bonferroni’s test for post-hoc comparisons. To determine the correlations between behavioral and biochemical variables, both the escape latency and number of error of the last probe day in the Barnes Maze test were used as behavioral variables. The correlations of behavioral variables in 24 mice with Sirt3 expression, Sirt3 deacetylation activity, MMP, ROS and OCR levels were evaluated by the Pearson correlation analysis. A *P*-value less than 0.05 was considered statistically significant. Prism 6 software (Graph Pad Software, Inc, La Jolla, CA) was used for the data analysis.

## Results

### Ginsenoside Rg1 attenuated I/S-induced neurocognitive disorders

**Figure 2:**
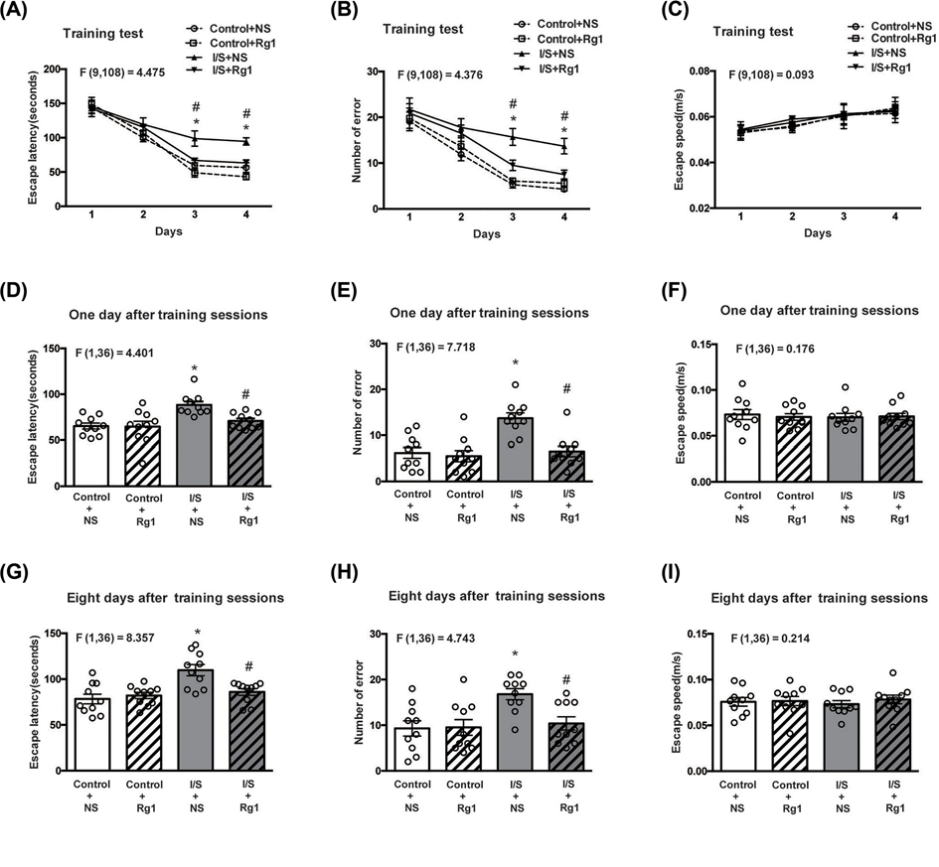
The influences of ginsenoside Rg1 treatment on neurocognitive disorders induced by I/S intervention Ginsenoside Rg1 treatment attenuated the prolongation of escape latency (**A,D,G**) and increase in number of errors (**B,E,H**) induced by I/S intervention but did not change mean speed (**C,F,I**) in the Barnes Maze test. *P<0.05 versus control + NS; ^#^*P*<0.05 versus I/S + NS. *n*=10. Data are presented as mean ± SEM.

As shown in Figure 2A,B, both the escape latency and number of errors on the third and fourth training days were significantly increased in the I/S+NS group compared with control+NS group. There were no obvious differences in the escape latency and number of errors between control+Rg1 and control+NS groups. However, deterioration of both the escape latency and number of errors on the third and fourth training days was evidently improved in the I/S+Rg1 group compared with I/S+NS group.

As shown in Figure 2D,E,G,H, both the escape latency and number of errors on the first and eighth days after training sessions were significantly increased in the I/S+NS group compared with control+NS group. There were no significant differences in the escape latency and number of errors between control+Rg1 and control+NS groups. However, deterioration of both the escape latency and number of errors on the first and eighth days after training sessions weres obviously attenuated in the I/S+Rg1 group compared with I/S+NS group. There were no significant differences in the escape speed during training and probe days among groups (Figure 2C,F,I).

### Ginsenoside Rg1 attenuated I/S-induced oxidative stress

**Figure 3:**
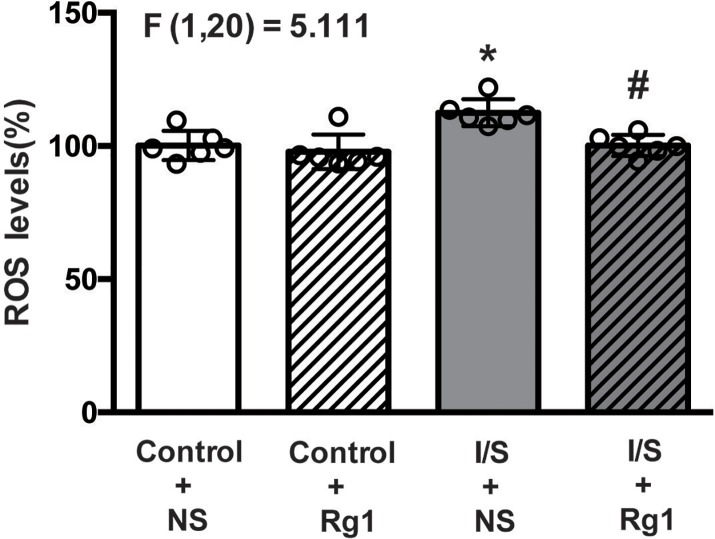
Ginsenoside Rg1 treatment alleviated the ROS elevation in hippocampus tissues induced by I/S intervention *P<0.05 versus control + NS; ^#^*P*<0.05 versus I/S + NS. *n*=6. Data are presented as mean ± SEM.

The ROS levels in the hippocampus tissues were shown in Figure 3. The ROS levels were significantly increased in the I/S+NS group (gray bar) compared with control+NS group (white bar). There was no significant difference in the ROS levels between control+Rg1 (white and striped bar) and control+NS (white bar) groups. However, ROS elevation was evidently alleviated in the I/S+Rg1 group (gray and striped bar) compared with I/S+NS group (gray bar).

### Ginsenoside Rg1 attenuated I/S-induced oxygen consumption reduction

**Figure 4:**
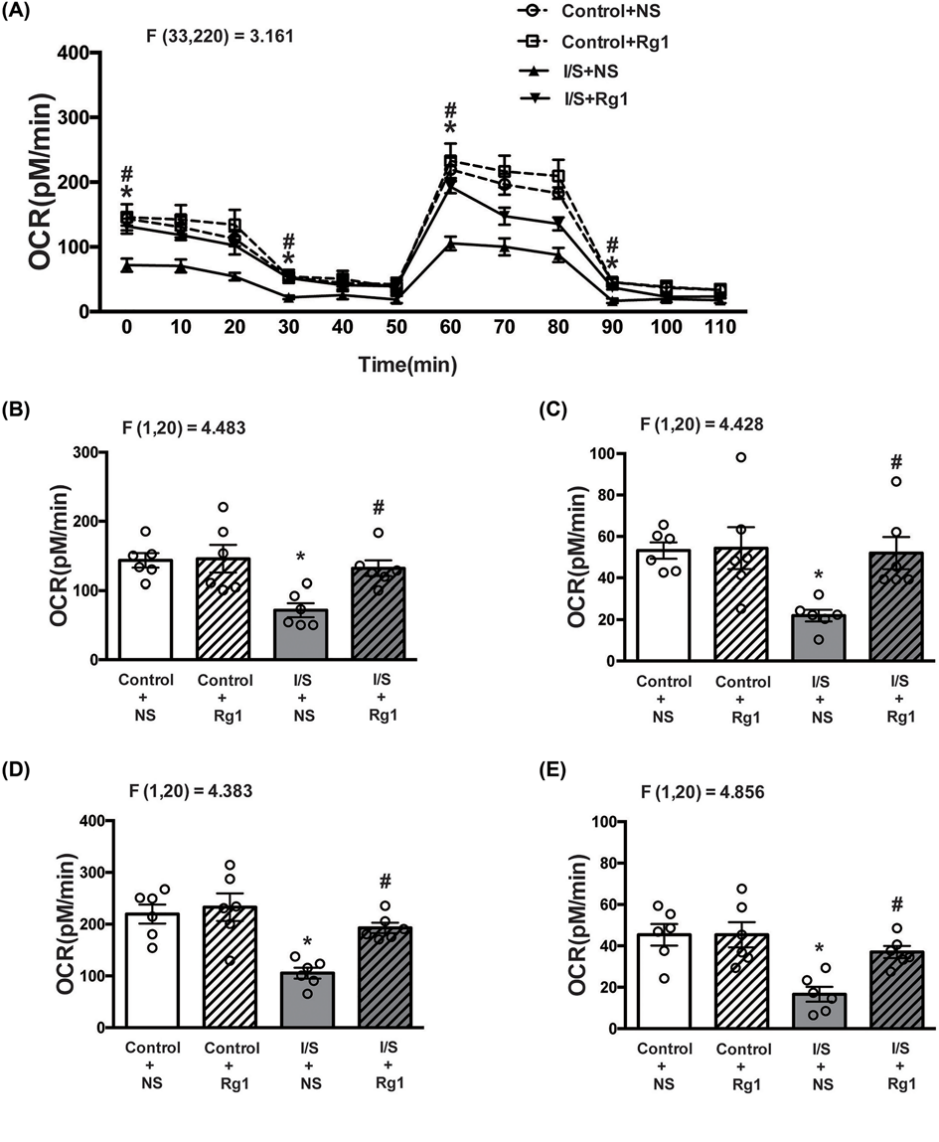
Ginsenoside Rg1 treatment abated the OCR reduction in hippocampus tissues induced by I/S intervention (**A**) The OCR curves; (**B**) basal OCR; (**C**) ATP level; (**D**) maximal mitochondrial respiratory capacity; (**E**) uncoupling capacity. *P<0.05 versus control + NS; ^#^*P*<0.05 versus I/S + NS. *n*=6. Data are presented as mean ± SEM.

As shown in Figure 4A, ginsenoside Rg1 treatment obviously mitigated the OCR reduction in the hippocampus tissues induced by I/S intervention. In particular, the basal mitochondrial respiration levels (OCR at 0 min) were significantly decreased in the I/S+NS group (gray bar, Figure 4B) compared with control+NS group (white bar, Figure 4B). There was no significant difference in the basal mitochondrial respiration levels between control+Rg1 (white and striped bar, Figure 4B) and control+NS (white bar, Figure 4B) groups. Compared with I/S+NS group (gray bar, Figure 4B), however, the decrease in the basal mitochondrial respiration levels was significantly attenuated in the I/S+Rg1 group (gray and striped bar, Figure 4B).

The ATP production (OCR at 30 min) was significantly decreased in the I/S+NS group (gray bar, Figure 4C) compared with control+NS group (white bar, Figure 4C). The ATP production did not obviously change in the control+Rg1 group (white and striped bar, Figure 4C) compared with control+NS group (white bar, Figure 4C), but was evidently abated in the I/S+Rg1 group (gray and striped bar, Figure 4C) compared with I/S+NS group (gray bar, Figure 4C). Furthermore, maximal mitochondrial respiratory capacity (OCR at 60 min) was significantly decreased in the I/S+NS group (gray bar, Figure 4D) compared with control+NS group (white bar, Figure 4D). The maximal mitochondrial respiratory capacity did not obviously change in the control+Rg1 group (white and striped bar, Figure 4D) compared with control+NS group (white bar, Figure 4D), but the decrease in the maximal mitochondrial respiratory capacity was significantly attenuated in the I/S+Rg1 group (gray and striped bar, Figure 4D) compared with I/S+NS group (gray bar, Figure 4D).

The uncoupling capacity (OCR at 90 min) was significantly decreased in the I/S+NS group (gray bar, Figure 4E) compared with control+NS group (white bar, Figure 4E). The uncoupling capacity did not obviously change in the control+Rg1 group (white and striped bar, Figure 4E) compared with control+NS group (white bar, Figure 4E), but the decrease in the uncoupling capacity was evidently improved in the I/S+Rg1 group (gray and striped bar, Figure 4E) compared with I/S + NS group (gray bar, Figure 4E).

### Ginsenoside Rg1 attenuated I/S-induced reduction in MMP

**Figure 5:**
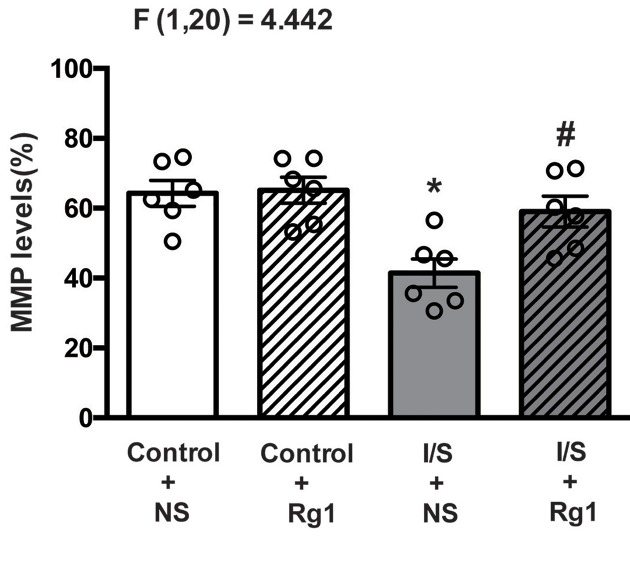
Ginsenoside Rg1 treatment attenuated the MMP reduction in hippocampus tissues induced by I/S intervention *P<0.05 versus control + NS; ^#^*P*<0.05 versus I/S + NS. *n*=6. Data are presented as mean ± SEM.

As shown in Figure 5, the MMP in the hippocampus tissues were significantly reduced in the I/S+NS group (gray bar) compared with control+NS group (white bar). The MMP did not obviously change in the control+Rg1 group (white and striped bar) compared with control+NS group (white bar), but the reduction in the MMP was significantly attenuated in the I/S+Rg1 group (gray and striped bar) compared with I/S+NS group (gray bar).

### Ginsenoside Rg1 attenuated I/S-induced reduction in Sirt3 expression and deacetylation activity

**Figure 6:**
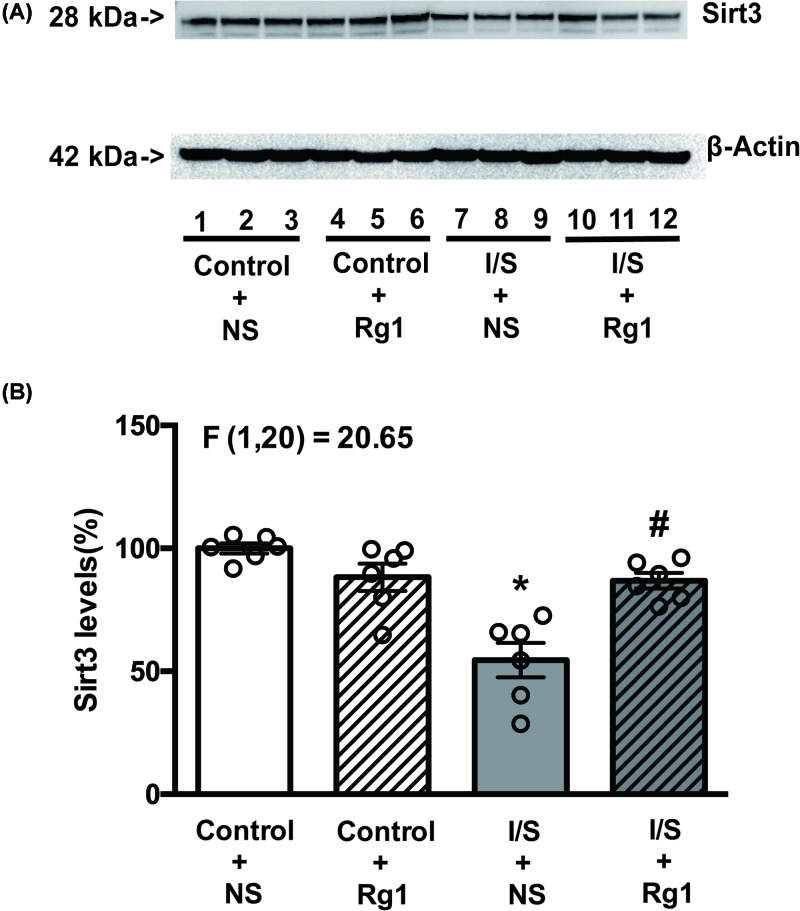
Ginsenoside Rg1 treatment alleviated the Sirt3 expression reduction in hippocampus tissues induced by I/S intervention *P<0.05 versus control + NS; ^#^*P*<0.05 versus I/S + NS. *n*=6. Data are presented as mean ± SEM.

**Figure 7:**
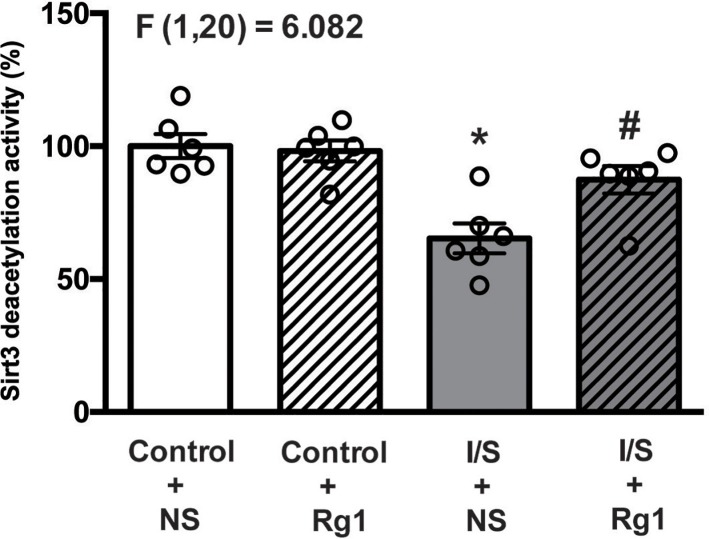
Ginsenoside Rg1 treatment mitigated the reduction in Sirt3 deacetylation activity in hippocampus tissues induced by I/S intervention *P<0.05 versus control + NS; ^#^*P*<0.05 versus I/S + NS. *n*=6. Data are presented as mean ± SEM.

As shown in Figures 6 and 7, both expression and deacetylation activity of Sirt3 in the hippocampus tissues were significantly reduced in the I/S+NS group (gray bar) compared with control+NS group (white bar). Both expression and deacetylation activity of Sirt3 did not obviously change in the control+Rg1 group (white and striped bar) compared with control+NS group (white bar), but the reduction in the expression and deacetylation activity of Sirt3 were significantly abated in the I/S+Rg1 group (gray and striped bar) compared with I/S+NS group (gray bar).

### Correlations between behavioral and biochemical variables

**Figure 8:**
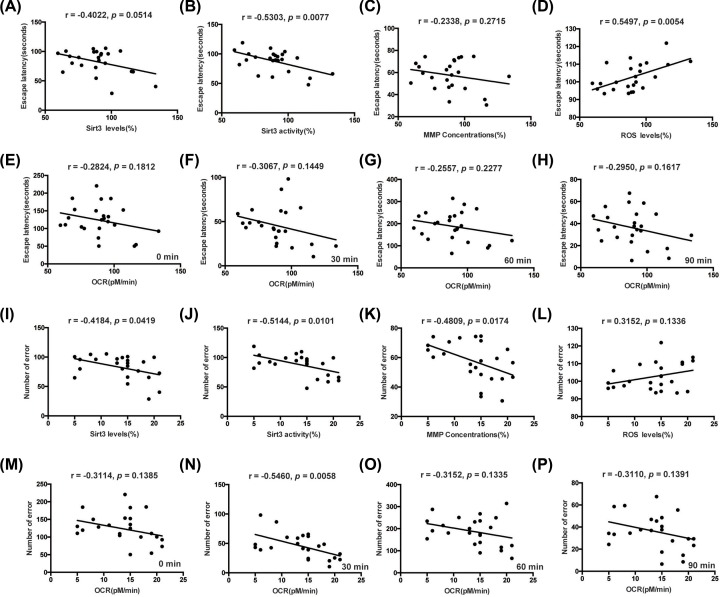
The correlations between behavioral and biochemical variables The correlations between escape latency or number of error of the last probe day in Barnes Maze and the Sirt3 expression (**A,I**), Sirt3 deacetylation activity (**B,J**), MMP (**C,K**), ROS (**D,L**) and OCR levels (**E**–**H,M**–**P**). *n*=24.

The correlations between behavioral and biochemical variables were shown in Figure 8A–P. The Sirt3 deacetylation activity was significantly correlated with both the escape latency and number of error (Figure 8B,J). The ROS levels were significantly correlated with the escape latency (Figure 8D). Furthermore, Sirt3 expression, MMP and OCR levels at 30 min were significantly correlated with the number of error (Figure 8I,K,N).

## Discussion

The present study was the first time to determine whether ginsenoside Rg1 treatment could provide a protection against the I/S-induced postoperative neurocognitive disorders in mice. Our results showed that the use of ginsenoside Rg1 treatment before surgery obviously ameliorated the I/S-induced neurocognitive disorders, as indicated by improvement on the prolonged escape latency and increased number of errors on the third and fourth training days in the Barnes maze test. Meanwhile, ginsenoside Rg1 treatment improved the I/S-induced memory disorders on the first and eighth days after training sessions. Our results are in agreement with the findings of previous studies regarding the influence of ginsenoside Rg1 treatment on the neurocognitive function in aged rats [13,21]. In the study of Alzheimer’s disease mice aged 6–9 months, Fang et al. [12] also showed that the use of ginsenoside Rg1 treatment for 3 months via intraperitoneal injection can significantly improve the learning and memory disorders. In the rats with chronic morphine administration, moreover, Qi et al. [22] demonstrate that ginsenoside Rg1 treatment may decrease the escape latency and increase the time spent in the platform quadrant and entering frequency in the Morris water maze test.

It is generally believed that mitochondrial dysfunction is one of important mechanisms of postoperative neurocognitive disorders [7]. The available literatures indicate that mitochondrial dysfunction may lead to the loss of synaptic markers like postsynaptic density-95 and synaptophysin or synaptic plasticity disorders [5,20]. Furthermore, mitochondria play a critical regulatory role in the presynaptic function of central nervous system [23]. It has been shown that anesthetics/surgery intervention may cause mitochondrial dysfunction, including the increased ROS production and the decreased respiration chain transmission like complex IV activity and ATP production [4–6]. In contrast, the ROS inhibitor EUK-134, NADPH oxidase inhibitor apocynin or mPTP opening inhibitor cyclosporine A may attenuate anesthetics/surgery intervention induced mitochondrial dysfunction, with an improved neurocognitive function [4,24,25]. Similarly, ginsenoside Rg1 has been demonstrated to attenuate mitochondrial dysfunction in many organs, such as brain, heart, liver and kidney [22,26–28]. The detailed mechanisms that ginsenoside Rg1 attenuates mitochondrial dysfunction remain unclear, but maybe involved in various pathways, including inhibition of oxidative stress and apoptosis; regulation of ROS generation, peroxisome proliferator-activated receptor γ and caspase-3 activation; affection of Akt/mTOR signal pathway and others [14,27]. Specially, increased ROS production, mainly released by the mitochondrial oxidative metabolism in certain pathological process, are likely attributable to inefficient mitochondrial respiration [29]. In addition, increased ROS production may also decrease the MMP and then aggravate the mitochondrial respiration chain dysfunction to generate more ROS, forming a vicious circle [30]. The present study showed that ginsenoside Rg1 treatment significantly ameliorated I/S-induced the evaluation of ROS production and the decline in OCR and MMP. According to results of previous and our studies, we deduce that mitochondrial pathway should be involved in the protection of ginsenoside Rg1 treatment against the I/S-induced postoperative neurocognitive disorders.

Increasing evidence has indicated the role of post-translational modifications of proteins, particularly acetylation, in neurodegenerative and cardiovascular diseases, diabetes, cancer and in aging [31]. Especially, acetylation of mitochondrial proteins has been shown to be involved in the pathogenesis of neurodegenerative diseases [32]. In fact, more than 60% of mitochondrial proteins contain acetylation sites, and most of these proteins are involved in the mitochondrial bioenergetics [31]. Thus, regulation of enzymatic deacetylation becomes one of the most important mechanisms controlling acetylation/deacetylation of mitochondrial proteins. It is usually considered that acetylation/deacetylation of mitochondrial proteins is a key regulator of mitochondrial metabolism and function. As a main mitochondrial sirtuin, moreover, Sirt3 has been proved to play a key role in maintaining metabolic and redox balance in the mitochondria under physiological and pathological conditions. Especially, Sirt3 regulates the enzymatic activity of proteins involved in fatty acid oxidation, tricarboxylic acid cycle, electron transport chain and oxidative phosphorylation [33–35]. Kong et al. [36] find that Sirt3 is essential for peroxisome proliferator-activated receptor γ co-activator-1α-dependent induction of ROS-detoxifying enzymes and several components of respiratory chain in human embryonic kidney cell lines. In the study of mice heart, Hafner et al. [9] demonstrate that Sirt3 can regulate the opening of mPTP by deacetylating acetylated cyclophilin D. Recently, Sirt3 has been associated with neurocognitive performance and pathological changes (amyloid-β and τ levels) in the mice model of Alzheimer’s disease [32]. Furthermore, Sirt3-knockout mice exhibit a poor remote memory, an impaired long-term potentiation and a decreased neuronal number in the anterior cingulate cortex [37]. In agreement with the findings of these studies, our experiment showed that I/S intervention resulted in the reduction in Sirt3 expression and deacetylation activity, while ginsenoside Rg1 treatment significantly ameliorated these changes of Sirt3 dysfunction caused by I/S intervention. By analyzing the correlations between behavioral performance and biochemical variables, our results showed that Sirt3 deacetylation activity was correlated well with both the escape latency and number of error, indicating that Sirt3 deacetylation activity as a biochemical marker may provide a useful reference for identification and treatment of I/S induced cognitive deficits. Accordingly, we infer that ginsenoside Rg1 treatment should provide a protection against I/S-induced neurocognitive disorders and improve Sirt3 dysfunction. Furthermore, ginsenoside Rg1 maybe a molecule that can be used as an agonist of Sirt3, targeting the energy metabolic disorders and oxidative stress of mitochondria for prevention of I/S-induced neurocognitive disorders.

There are several limitations in our experiment. First, only a single dosage regimen of ginsenoside Rg1 treatment was selected according to available literature and the effects of different-dose ginsenoside Rg1 treatments on I/S-induced neurocognitive disorders were not assessed. Thus, it is unclear whether protective effect of ginsenoside Rg1 treatment on I/S-induced neurocognitive disorders is dose-independent and has a ceiling effect. Second, this experiment only used the Barnes maze test to assess the neurocognitive function. Thus, it is not determined whether ginsenoside Rg1 treatment can significantly improve I/S-induced neurocognitive disorders by other behavioral assessment methods, especially the use of the Morris Water Maze test or Fear Condition System to validate if ginsenoside Rg1 treatment may improve certain different domains of I/S-induced neurocognitive disorders. Third, the present study only focused the changes of Sirt3 expression and deacetylation activity, but did not reveal the special proteins interacted with Sirt3, as there have been many substrates that may interact with Sirt3 and the proving test is ongoing. Thus, further experiments are still needed to address these issues.

## Conclusions

This experiment demonstrates that ginsenoside Rg1 treatment can significantly ameliorate I/S-induced neurocognitive disorders and Sirt3 dysfunction. Thus, ginsenoside Rg1 maybe a small molecule natural compound as Sirt3 agonist and has the potential implication for treatment of I/S-induced neurocognitive disorders pending future studies.

## Abbreviations

H4-APP: stably transfected H4 human neuroglioma cells that express full-length human amyloid precursor protein
I/S: isoflurane/surgery
MMP: mitochondrial membrane potential
mPTP: mitochondrial permeability transition pore
NS: normal saline
OCR: oxygen consumption rate
PND: perioperative neurocognitive disorder
ROS: reactive oxygen species
Sirt3: Sirtuin3

## References

[1] Evered L., Silbert B., Knopman D.S., Scott D.A., DeKosky S.T., Rasmussen L.S. et al. (2018) Recommendations for the nomenclature of cognitive change associated with anaesthesia and surgery. Br. J. Anaesth. 121, 1005–1012 10.1016/j.bja.2017.11.087 30336844 PMC7069032

[2] Moller J.T., Cluitmans P., Rasmussen L.S., Houx P., Rasmussen H., Canet J. et al. (1998) Long-term postoperative cognitive dysfunction in the elderly: ISPOCD1 study. The Lancet 351, 857–861 10.1016/S0140-6736(97)07382-0 9525362

[3] Caja S.and Enriquez J.A. (2017) Mitochondria in endothelial cells: sensors and integrators of environmental cues. Redox. Biol. 12, 821–827 10.1016/j.redox.2017.04.021 28448943 PMC5406579

[4] Peng M., Zhang C., Dong Y., Zhang Y., Nakazawa H., Kaneki M. et al. (2016) Battery of behavioral tests in mice to study postoperative delirium. Sci. Rep. 6, 29874 10.1038/srep29874 27435513 PMC4951688

[5] Miao H., Dong Y., Zhang Y., Zheng H., Shen Y., Crosby G. et al. (2018) Anesthetic isoflurane or desflurane plus surgery differently affects cognitive function in Alzheimer’s disease transgenic mice. Mol. Neurobiol. 55, 5623–5638 10.1007/s12035-017-0787-9 28986748 PMC5889364

[6] Miao H., Zhen Y., Ding G., Hong F., Xie Z.and Ming T. (2015) Ginsenoside Rg1 attenuates isoflurane-induced caspase-3 activation via inhibiting mitochondrial dysfunction. Biomed. Environ. Sci. 28, 116–126 25716562 10.3967/bes2015.014

[7] Vutskits L.and Xie Z. (2016) Lasting impact of general anaesthesia on the brain: mechanisms and relevance. Nat. Rev. Neurosci. 17, 705–717 10.1038/nrn.2016.128 27752068

[8] Yin X., Li S., Nielsen M., Carcione T., Liang W.and Jiong S. (2018) Sirtuin 3 attenuates amyloid-β induced neuronal hypometabolism. Aging 10, 2874–2883 10.18632/aging.101592 30362958 PMC6224231

[9] Hafner A., Dai J., Gomes A., Xiao C., Palmeira K., Rosenzweig A. et al. (2010) Regulation of the mPTP by SIRT3-mediated deacetylation of CypD at lysine 166 suppresses age‐related cardiac hypertrophy. Aging 2, 914–923 10.18632/aging.100252 21212461 PMC3034180

[10] Ye J., Yao J.P., Wang X., Zheng M., Li P., He C. et al. (2016) Neuroprotective effects of ginsenosides on neural progenitor cells against oxidative injury. Mol. Med. Rep. 13, 3083–3091 10.3892/mmr.2016.4914 26935530 PMC4805061

[11] Zhang X., Wang J., Xing Y., Gong L., Li H., Wu Z. et al. (2012) Effects of ginsenoside Rg1 or 17β-estradiol on a cognitively impaired, ovariectomized rat model of Alzheimer’s disease. Neuroscience 220, 191–200 10.1016/j.neuroscience.2012.06.027 22728092

[12] Fang F., Chen X., Huang T., Lue L.F., Luddy J.S.and Yan S.S. (2012) Multi-faced neuroprotective effects of Ginsenoside Rg1 in an Alzheimer mouse model. Biochim. Biophys. Acta 1822, 286–292 10.1016/j.bbadis.2011.10.004 22015470 PMC3304026

[13] Chen L., Yao H., Chen X., Wang Z., Xiang Y., Xia J. et al. (2018) Ginsenoside Rg1 decreases oxidative stress and down-regulates Akt/mTOR signalling to attenuate cognitive impairment in mice and senescence of neural stem cells induced by D-Galactose. Neurochem. Res. 43, 430–440 10.1007/s11064-017-2438-y 29147958

[14] Chen L.M., Lin Z.Y., Zhu Y.G., Lin N., Zhang J., Pan X.D. et al. (2012) Ginsenoside Rg1 attenuates β-amyloid generation via suppressing PPARγ-regulated BACE1 activity in N2a-APP695 cells. Eur. J. Pharmacol. 675, 15–21 10.1016/j.ejphar.2011.11.039 22166376

[15] Fan C., Song Q., Wang P., Li Y., Yang M.and Yu S.Y. (2018) Neuroprotective effects of ginsenoside-Rg1 against depression-like behaviors via suppressing glial activation, synaptic deficits, and neuronal apoptosis in rats. Front. Immunol. 9, 2889 10.3389/fimmu.2018.02889 30581440 PMC6292928

[16] Zhang J., Jiang W.and Zuo Z. (2014) Pyrrolidine dithiocarbamate attenuates surgery-induced neuroinflammation and cognitive dysfunction possibly via inhibition of nuclear factor κB. Neuroscience 26, 1–10 10.1016/j.neuroscience.2013.12.034 PMC3950371 24365462

[17] Tan H., Bi J., Wang Y., Zhang J.and Zuo Z. (2015) Transfusion of old RBCs induces neuroinflammation and cognitive impairment. Crit. Care Med. 43, e276–e286 10.1097/CCM.0000000000001023 25860202 PMC4506264

[18] Mu Q., Fang X., Li X., Zhao D., Mo F., Jiang G. et al. (2015) Ginsenoside Rb1 promotes browning through regulation of PPARγ in 3T3-L1 adipocytes. Biochem. Biophys. Res. Commun. 466, 530–535 10.1016/j.bbrc.2015.09.064 26381176

[19] Ju X., Wen Y., Metzger D.and Jung M. (2013) The role of p38 in mitochondrial respiration in male and female mice. Neurosci. Lett. 544, 152–156 10.1016/j.neulet.2013.04.004 23603578 PMC3698872

[20] XuG., LuH., DongY., ShapovalD., SorianoS.G., LiuX.et al. (2017) Coenzyme Q10 reduces sevoflurane-induced cognitive deficiency in young mice. Br. J. Anaesth. 119, 481–491 10.1093/bja/aex071 28482003

[21] Zhang Y., Zhang Z., Wang H., Cai N., Zhou S., Zhao Y. et al. (2016) Neuroprotective effect of ginsenoside Rg1 prevents cognitive impairment induced by isoflurane anesthesia in aged rats via antioxidant, anti-inflammatory and anti-apoptotic effects mediated by the PI3K/AKT/GSK-3β pathway. Mol. Med. Rep. 14, 2778–2784 10.3892/mmr.2016.5556 27485139

[22] Qi D., Zhu Y., Wen L., Liu Q.and Qiao H. (2009) Ginsenoside Rg1 restores the impairment of learning induced by chronic morphine administration in rats. J. Psychopharmacol. 23, 74–83 10.1177/0269881107082950 18308784

[23] Rangaraju V., Calloway N.and Ryan T.A. (2014) Activity-driven local ATP synthesis is required for synaptic function. Cell 156, 825–835 10.1016/j.cell.2013.12.042 24529383 PMC3955179

[24] Sun Z., Satomoto M., Adachi Y.U., Kinoshita H.and Makita K. (2016) Inhibiting NADPH oxidase protects against long-term memory impairment induced by neonatal sevoflurane exposure in mice. Br. J. Anaesth. 117, 80–86 10.1093/bja/aew064 27147542 PMC4913390

[25] Boscolo A., Starr J.A., Sanchez V., Lunardi N., DiGruccio M.R., Ori C. et al. (2012) The abolishment of anesthesia-induced cognitive impairment by timely protection of mitochondria in the developing rat brain: the importance of free oxygen radicals and mitochondrial integrity. Neurobiol. Dis. 45, 1031–1041 10.1016/j.nbd.2011.12.022 22198380 PMC3276740

[26] Korivi M., Hou C.W., Huang C.Y., Lee S.D., Hsu M.F., Yu S.H. et al. (2012) Ginsenoside-Rg1 protects the liver against exhaustive exercise-induced oxidative stress in rats. Evid. Based Complement. Alternat. Med. 2012, 932165 10.1155/2012/932165 21941591 PMC3176525

[27] Qin L., Fan S., Jia R.and Liu Y. (2018) Ginsenoside Rg1 protects cardiomyocytes from hypoxia-induced injury through the PI3K/AKT/mTOR pathway. Die Pharmazie 73, 349–355 29880088 10.1691/ph.2018.8329

[28] Li S.S., He A.L., Deng Z.Y.and Liu Q.F. (2018) Ginsenoside-Rg1 protects against renal fibrosis by regulating the Klotho/TGF-β1/Smad signaling pathway in rats with obstructive nephropathy. Biol. Pharm. Bull. 41, 585–591 10.1248/bpb.b17-00934 29607931

[29] Khacho M., Tarabay M., Patten D., Khacho P., MacLaurin J.G., Guadagno J. et al. (2014) Acidosis overrides oxygen deprivation to maintain mitochondrial function and cell survival. Nat. Commun. 5, 3550 10.1038/ncomms4550 24686499 PMC3988820

[30] Moreira P.I., Carvalho C., Zhu X., Smith M.A.and Perry G. (2010) Mitochondrial dysfunction is a trigger of Alzheimer’s disease pathophysiology. Biochim. Biophys. Acta 1802, 2–10 10.1016/j.bbadis.2009.10.006 19853658

[31] Parodi-Rullan R.M., Chapa-Dubocq X.R.and Javadov S. (2018) Acetylation of mitochondrial proteins in the heart: the role of SIRT3. Front. Physiol. 9, 1094 10.3389/fphys.2018.01094 30131726 PMC6090200

[32] Yin J., Han P., Song M., Nielsen M., Beach T.G, Serrano G.E et al. (2018) Amyloid-β increases tau by mediating sirtuin 3 in Alzheimer’s disease. Mol. Neurobiol. 55, 8592–8601 10.1007/s12035-018-0977-0 29574628 PMC6677544

[33] Koentges C., Pfeil K., Schnick T., Wiese S., Dahlbock R., Cimolai M.C. et al. (2015) SIRT3 deficiency impairs mitochondrial and contractile function in the heart. Basic Res. Cardiol. 110, 36 10.1007/s00395-015-0493-6 25962702

[34] Dittenhafer-Reed K.E., Richards A.L., Fan J., Smallegan M.J., Fotuhi Siahpirani A., Kemmerer Z.A. et al. (2015) SIRT3 mediates multi-tissue coupling for metabolic fuel switching. Cell Metab. 21, 637–646 10.1016/j.cmet.2015.03.007 25863253 PMC4393847

[35] Fan W.and Evans R. (2015) PPARs and ERRs: molecular mediators of mitochondrial metabolism. Curr. Opin. Cell. Biol. 33, 49–54 10.1016/j.ceb.2014.11.002 25486445 PMC4380823

[36] Kong X., Wang R., Xue Y., Liu X., Zhang H., Chen Y. et al. (2010) Sirtuin 3, a new target of PGC-1α, plays an important role in the suppression of ROS and mitochondrial biogenesis. PLoS ONE 5, e11707 10.1371/journal.pone.0011707 20661474 PMC2908542

[37] Kim H., Kim S., Choi J.E., Han D., Koh S.M., Kim H.S. et al. (2019) Decreased neuron number and synaptic plasticity in SIRT3-knockout mice with poor remote memory. Neurochem. Res. 44, 676–682 10.1007/s11064-017-2417-3 29076061

